# Les invaginations intestinales chez l’adulte: à propos de 17 cas

**Published:** 2012-06-01

**Authors:** Elhattabi Khalid, Bensardi Fatimazahra, Khaiz Driss, Fadil Abdelaziz, Raouah Abdellatif, Lefriyekh Rachid, Benissa Nadia, Berrada Saad, Zerouali Ouariti Najib

**Affiliations:** 1 Service des urgences chirurgicales viscérales – CHU Ibn Rochd, Casablanca, Maroc

**Keywords:** Invagination, douleur, scanner, chirurgie

## Abstract

L’invagination intestinale est une affection rare chez l’adulte. Elle conduit le plus souvent à la découverte d’une cause organique pouvant être tumorale. Le but de notre travail est de dégager les particularités épidémiologiques diagnostiques et thérapeutiques de cette affection; à travers une étude descriptive rétrospective, ayant portée sur 17 cas d’invagination intestinale de l’adulte opérés dans le service des Urgences Chirurgicales Viscérales du CHU Ibn Rochd de Casablanca du 1er janvier 2006 au 31 décembre 2010. La douleur abdominale était présente chez tout les patients; L’échographie abdominale pratiquée chez 12 patients; elle a montré une image en cocarde dans 9 cas, une masse abdominale dans 1 cas et un épaississement grèlique dans 5 cas. La tomodensitométrie abdominale faite chez 15 patients a objectivé l’invagination intestinale dans tous les cas. Le traitement chirurgical a été adopté chez tous les patients; il a permis de faire la résection des segments intestinaux invaginé dans tout les cas. Le résultat anatomopathologique de la pièce de résection a retrouvé une cause organique de l’invagination dans dix cas (58,8%). L’invagination intestinale chez l’adulte est souvent secondaire à une lésion organique : tumorale ou inflammatoire. Elle se caractérise par son polymorphisme clinique. Il s’agit essentiellement de phénomènes subocclusifs à répétition. Concernant le traitement de l’invagination intestinale de l’adulte, la résection du segment invaginé est toujours nécessaire car dans 80% des cas, cette affection est secondaire à une lésion organique qui doit être traitée.

## Introduction

L’invagination intestinale est définie par la pénétration ou le télescopage d’un segment intestinal dans celui situé immédiatement en aval. C’est une affection rare chez l’adulte qui représente 1 à 5 % des occlusions intestinales [1, 2];Par opposition aux formes de l’enfant qui sont dans 90% des cas primitives, les invaginations intestinales de l’adulte conduisent souvent à la découverte d’une cause organique pouvant être tumorale ou non [1].

Le but de notre travail est de dégager les particularités épidémiologiques diagnostiques et thérapeutiques de cette affection; à travers une étude rétrospective descriptive de 17 cas d′invagination intestinale de l′adulte, colligés dans le service des Urgences Chirurgicales Viscérales du CHU Ibn Rochd de Casablanca.

## Methods

Il s’agit d’une étude rétrospective descriptive, ayant porté sur 17 cas d’invagination intestinale de l’adulte opérés dans le service des Urgences Chirurgicales Viscérales du CHU Ibn Rochd de Casablanca du 1er janvier 2006 au 31 décembre 2010.

## Résultats

En 5 ans, 528 occlusions mécaniques ont été recensées, dont 17 cas d’invaginations intestinales chez l′adulte (soit 3.21 %). L′âge moyen était de 41 ans, avec des extrêmes de 17 et 70 ans. Une prédominance masculine a été noté avec 11 hommes et 6 femmes; avec un sex-ratio de 1.8.

Sur le plan clinique; la douleur abdominale était présente chez tout les patients; huit entre eux (47%) ont été reçus dans un tableau d′occlusion intestinale et trois patients avaient un tableau chronique fait de douleurs abdominales et de constipation avec altération de l′état général .Un seul malade avait des rectorragies . A l’examen clinique le boudin d’invagination a été palpé chez trois patients.

**Figure 1:**
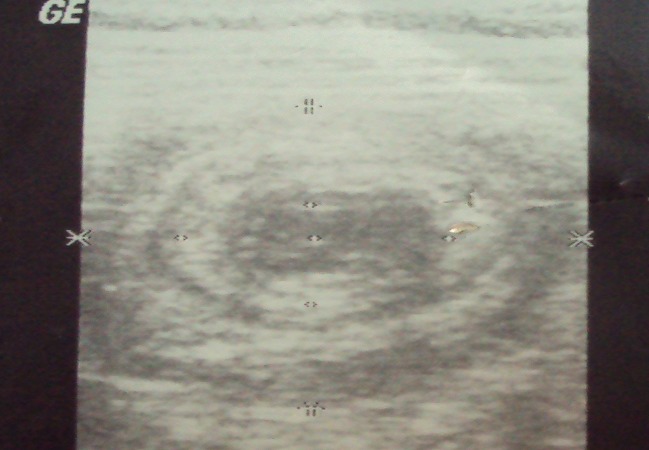
Image en cocarde à l’échographie abdominale

**Figure 2:**
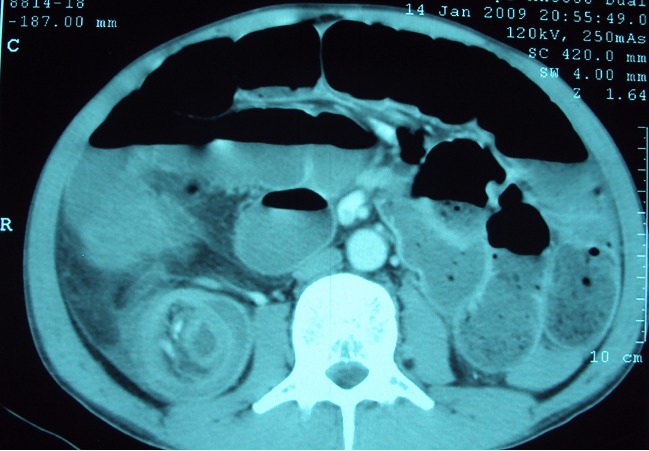
Anneaux concentriques hypo ou hyperdenses (aspect en cocarde ou cible) au scanner abdominal

**Figure 3:**
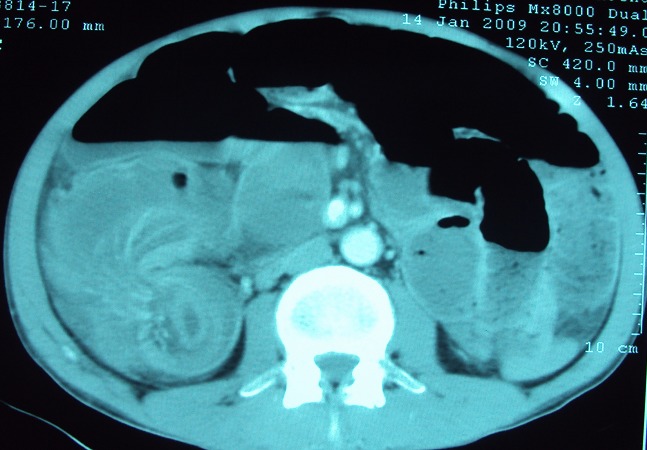
Image en croissant avec mésentère invaginé au scanner abdominal

La radiographie de l′abdomen sans préparation, pratiquée chez tout les patients a révélé des niveaux hydro-aériques dans 7cas. L′échographie abdominale pratiquée chez 12 patients; elle a montré une image en cocarde dans 9 cas (Figure 1) , une masse abdominale dans 1 cas et un épaississement grèlique dans 5 cas. La tomodensitométrie abdominale faite chez 15 patients a objectivé l’invagination intestinale dans tous les cas (Figure 2, Figure 3).

**Figure 4:**
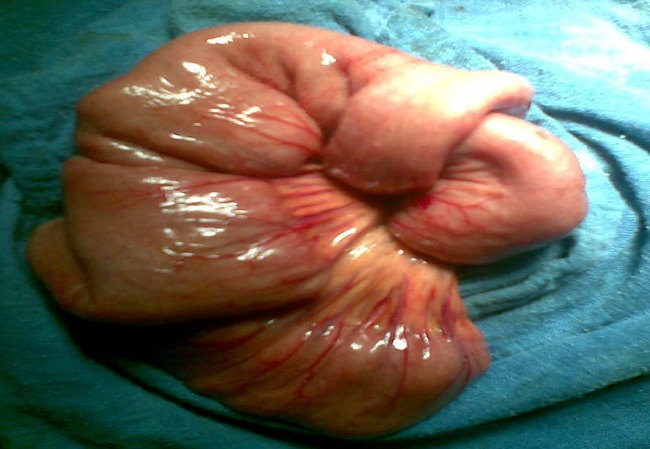
Vue per opératoire d’invagination grêlo-grêlique

**Figure 5:**
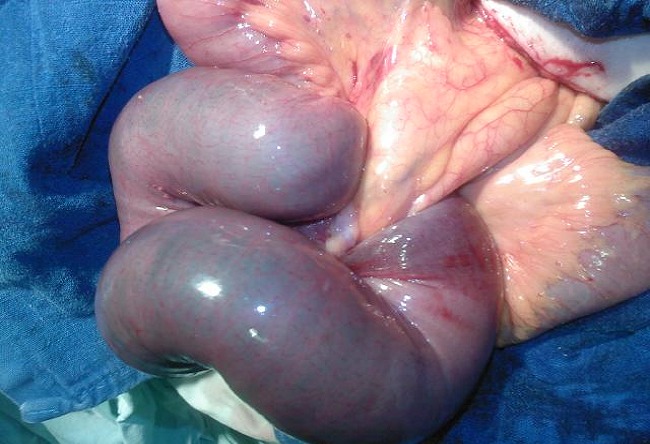
Vue per opératoire d’invagination grêlo-grêlique

Le traitement chirurgical a été adopté chez tout les patients par une voie d’abord conventionnelle (Table 1); il a permet de faire la résection des segments intestinaux invaginé dans tout les cas (Figure 4, Figure 5) . L’anastomose a été déférée chez deux patients qui étaient en péritonite suite à une nécrose intestinale.

**Table 1:** Les gestes chirurgicaux pratiqués

Type d’intervention	Nombre de cas	Pourcentage (%)
Résection grêlique	8	47,05
Résection grêlique + anastomose grêlo-grêlique termino-terminale	7	41,17
Résection iléale+ iléostomie en en canon de fusil	1	5,88
Résection iléo-colique	8	47,05
Hémicolectomie droite + anastomose iléo-colique termino-terminale	7	41,17
Hémicolectomie droite+ anastomose iléo-colique termino-latérale	1	5,88
Résection colique		
Résection colique segmentaire gauche+ colostomie iliaque gauche sur baguette	1	5,90
**Total**	**17**	**100**

Les suites opératoires ont été marquées par une péritonite secondaire à une désunion anastomotique dans un cas. Ce patient a bénéficié d′une réintervention avec iléo colostomie temporaire puis rétablissement de la continuité 4 mois plus tard. aucun cas de décès post opératoire n’a été noté dans notre série.

Le résultat anatomo pathologique de la pièce de résection a retrouvé une cause organique de l’invagination dans dix cas (58, 8%) (Table 2).

**Table 2:** Résultats anatomopathologique

Résultats	Nombres de cas	Pourcentage (%)
Lipome grêlique	3	17,65
Polype adénomateux	1	5,88
Polype inflammatoire non adénomateux	1	5,88
Adhérences	1	5,88
Lymphome de Malt iléale	2	11,76
Adénocarcinome iléal	2	11,76
Infarcissement de la paroi intestinale en rapport avec invagination, sans autre lésion organique	7	41,19
Total	17	100

## Discussion

l’invagination intestinale représente 1 à 5% des étiologies d’occlusions intestinales chez l’adulte, et 0,003 à 0,02% des hospitalisations ou une cause organique est trouvée dans 70 à 90% des cas et idiopathique dans 8 à 20% alors que, chez l’enfant l’invagination intestinale est primitive dans 90% cas [7–10].

Si cette affection ne s’observe que très rarement dans les pays développés, elle est au contraire relativement fréquente en Afrique et notamment en zones intertropicales. Les raisons de ces différences géographiques sont inconnues et certains facteurs tels que la diététique et les parasites sont évoqués [11] .

Il est difficile de retrouver une prédominance liée au sexe ou à une tranche d’âge; même si l’âge moyen des différentes séries publiées se situe entre 40 et 50 ans avec des extrêmes allant de 15 ans à 81 ans [5, 12, 13].

La symptomatologie clinique est polymorphe et le plus souvent trompeuse: tableau occlusif aigu, tableau subocclusif de survenue progressive s’étendant de quelques jours à quelques semaines, syndromes abdominaux non spécifiques (modification du transit, Douleurs abdominales diffuses, saignements digestifs?), évoluant Parfois pendant plusieurs mois, avec ou sans altération de l’état général [14–17].

La constatation à l’examen physique du malade d’une masse abdominale est un signe de grande valeur en particulier, si elle apparaît de siège et de consistance différents au cours d’examens répétés. Une fois sur deux en moyenne lorsqu’on est appelé à voir le malade en pleine crise, si le pannicule adipeux et le ballonnement abdominal ne sont pas trop importants, et si le relâchement musculaire de la paroi est suffisant, on sentira la tuméfaction correspondante au boudin d’invagination .On le cherchera soigneusement en décubitus latéral droit et gauche, en décubitus dorsal et en position de Trendelenburg [18–20] .

La palpation sous anesthésie générale juste avant l’opération a souvent permis la détection d’une masse abdominale jusque là non perçue, manœuvre à ne pas négliger car elle simplifie l’exploration chirurgicale . Ainsi on conçoit l’importance d’un examen physique complet de l’abdomen (associant palpation profonde et toucher rectal) pendant et entre les crises douloureuses plusieurs fois, de façon soigneuse et méthodique. A noter, qu’il ne faut pas confondre la masse correspondante au boudin d’invagination, avec une masse liée à l’affection étiologique (tumeur maligne ou bénigne)[21, 22]. La masse abdominale correspondant au boudin d’invagination est présente dans 24 à 42%, mais sa fréquence reste variable suivant les séries, du fait de son caractère fugace [5, 7, 13].

La perception du boudin au toucher rectal dépend de la longueur de l’invagination. Le doigt peut ramener des glaires sanguinolentes affirmant la rectorragie qui est un excellent signe de souffrance intestinale.

Sur la radiographie de l’abdomen sans préparation (ASP), une invagination iléo-colique ou iléo-iléale peut être soupçonné devant une opacité arrondi homogène de tonalité hydrique circonscrite sur un coté par un croissant clair et qui peut renfermer en son sein des images claires arciformes qui lui confèrent un aspect en «ressort à boudin» [23].

A l’échographie abdominale les signes typiques du boudin d’invagination correspondent à la visualisation des couches successives de parois digestives des anses invaginées et de l’anse receveuse avec au centre, un peu excentrée, la graisse du mésentère emportée par l’anse invaginée .En coupe transversale;l’image est en cocarde, faite d’une couronne périphérique plutôt hypoéchogène constituée de plusieurs couches digestives et comportant un croissant hyperéchogène excentré correspondant au mésentère incarcéré [24, 25] .En coupe longitudinale; l’image dite en sandwich ou en pseudo-rein, correspond à la succession des couches de paroi digestive hypoéchogène par rapport à la graisse mésentérique plus centrale et hyperéchogène. La zone de pénétration de l’anse invaginée dans l’anse réceptive peut être parfaitement visualisée [24].

L’échographie abdominale associée au doppler couleur peut dans certains cas mettre en évidence la disparition de l’hyperémie veineuse et artérielle du boudin d’invagination évocatrice de nécrose ischémique [26, 27].

La tomodensitométrie a connu un succès rapidement croissant dans l’exploration des douleurs abdominales aiguës chirurgicales en général, et de l’occlusion en particulier [28–30] . Elle permet de mettre en évidence l’invagination intestinale, avec une masse tissulaire correspondant à l’anse invaginée, accompagnée d’une image en croissant, excentrée de densité graisseuse en rapport avec le mésentère. Le corps de l’invagination se présente sous forme de multiples anneaux concentriques hypo ou hyperdenses, donnant un aspect en cocarde ou cible sur les coupes de face et en sandwich sur les coupes transversales [23, 31–33] .Elle permet également d’apprécier le degré de la souffrance viscérale [30, 34] .

Chez l’adulte, le traitement d’une invagination est toujours chirurgical, la plupart des auteurs admettent la nécessité d’une laparotomie exploratrice. Il n’y a en effet pas de place pour la réduction par hyperpression sous contrôle radiologique étant donnée la fréquence des formes d’étiologie tumorale [35, 36] .en effet dans les invaginations colo-coliques ou iléo-coliques: en raison de la fréquence du cancer en tant que lésion causale, la résection première (colectomie droite ou gauche) est préconisée par la majorité des auteurs, afin de limiter les risques de dissémination métastatique. Tandis que dans les invaginations du grêle, en présence d’un long segment intestinal invaginé, il parait licite de tenter une réduction préalable à fin de limiter l’étendue de l’exérèse car, à ce niveau, les tumeurs malignes sont rares.

La c’lioscopie constitue actuellement un véritable moyen de diagnostic et parfois de traitement de l’invagination intestinale du grêle [37].en cas d’occlusion intestinale elle nécessite une expertise en chirurgie laparoscopique du fait de la distension des anses grêles gênant la vision et rendant difficile leur mobilisation avec un risque élevé de plaies iatrogènes.

## Conclusion

L’invagination intestinale chez l’adulte est souvent secondaire à une lésion organique: tumorale ou inflammatoire. Elle se caractérise par son polymorphisme clinique. Il s’agit essentiellement de phénomènes subocclusifs à répétition. Concernant le traitement de l’invagination intestinale de l’adulte, la résection du segment invaginé est toujours nécessaire car cet accident n’est qu’un épiphénomène à la base duquel se trouve dans 80% des cas une lésion organique qui doit être traitée.
